# Clinical and immunological features of a patient exhibiting delayed puberty, microcephaly, scoliosis and epilepsy caused by a novel mutation in *IGSF10*

**DOI:** 10.3389/fcell.2026.1816964

**Published:** 2026-07-30

**Authors:** Gao-Hui Cao, Lv Liu, Zhen Liu, Lei Zhu, Na Ta, Yi-Hui Liu, Ai-Qian Zhang, Liang-Liang Fan

**Affiliations:** 1 Department of Obstetrics and Gynecology, The Third Xiangya Hospital, Central South University, Changsha, Hunan, China; 2 Department of Cell Biology, School of Life Science, Central South University, Changsha, Hunan, China; 3 Department of Respiratory Medicine, Second Xiangya Hospital of Central South University, Research Unit of Respiratory Disease of Central South University, Clinical Medical Research Center for Pulmonary and Critical Care Medicine in Hunan Province, Diagnosis and Treatment Center of Respiratory Disease in Hunan Province, Hunan Province Evidence-Based Medicine Center, Changsha, Hunan, China; 4 Department of Neurology, Affiliated Hospital of Yangzhou University, Yangzhou University, Yangzhou, Jiangsu, China; 5 Department of Obstetrics and Gynecology, Ordos Central Hospital, Ordos, Inner Mongolia, China

**Keywords:** delayed puberty, IGSF10, microcephaly, scoliosis, single-cell RNA sequencing

## Abstract

Members of the immunoglobulin superfamily (IgSF) are cell-surface proteins essential for a wide range of biological processes, including molecular transport, morphoregulation, and cell differentiation. IGSF10, a member of this family, has been identified as a disease-associated gene in delayed puberty and cleidocranial dysplasia. In this study, a Chinese patient with delayed puberty, microcephaly, scoliosis, and epilepsy was investigated. The genetic defect and immune alterations were assessed using whole-exome sequencing, Sanger sequencing, and single-cell RNA sequencing. After variant filtering, a novel IGSF10 variant, NM_178,822.5:c.4518del (p.His1506Gln fs Ter13), was identified in the proband. Sanger sequencing indicated that this variant was *de novo*, as it was present only in the proband and absent from her parents. Quantitative real-time PCR showed that the newly identified variant was associated with reduced IGSF10 mRNA abundance, consistent with nonsense-mediated decay. Single-cell RNA sequencing of peripheral blood mononuclear cells revealed that this variant was associated with marked alterations in effector T-cell and monocyte populations. Further analysis suggested altered differentiation patterns of T cells and monocytes, supporting the hypothesis that immune perturbations may contribute to the patient’s reproductive, neurological, and skeletal phenotypes, although this possibility requires functional validation. A novel IGSF10 variant was identified in a Chinese patient with delayed puberty, microcephaly, scoliosis, and epilepsy. This case expands the mutational spectrum of IGSF10 deficiency and suggests that its phenotypic spectrum may include scoliosis and epilepsy, pending further confirmation. These findings also provide preliminary insight into the potential interplay among IGSF10, immune function, hormone secretion, and brain and skeletal development.

## 1 Introduction

The immunoglobulin superfamily (IgSF) comprises a large group of proteins that are widely expressed on the plasma membranes of most cell types and are characterized by immunoglobulin or immunoglobulin-like domains at the extracellular N-terminus (Zinn and Ozkan, 2017; Verschueren et al., 2020). Because Ig domains mediate molecular recognition, binding, and intercellular interactions, IgSF members have been implicated in molecular transport, morphoregulation, and cell differentiation, and they play important roles in neurodevelopmental diseases, chronic diseases, immune disorders, and multiple cancers (Gennarini et al., 2017; Hagiyama et al., 2019; Odales et al., 2020; Jimbo et al., 2022; Li et al., 2022). As an IgSF member, IGSF10 is an essential gene involved in cell differentiation and developmental processes. In 2016, Howard et al. first reported that mutations in *IGSF10* can cause delayed puberty (Howard et al., 2016). Animal and cellular models showed that reduced IGSF10 may impair gonadotropin-releasing hormone (GnRH)-associated neuronal migration, potentially leading to functional hypogonadotropic hypogonadism (Howard et al., 2016). Subsequent large-scale screening of patients with hypogonadotropic hypogonadism also identified pathogenic IGSF10 mutations (Howard et al., 2016; Amato et al., 2019). In 2019, Budny et al. reported that IGSF10 mutations may also contribute to combined pituitary hormone deficiency (Budny et al., 2020). In 2021, Liu et al. described a Chinese family with cleidocranial dysplasia caused by an IGSF10 mutation (Liu et al., 2021). Functional assays showed that IGSF10 can modulate the onset of osteogenic differentiation through direct regulation of bone sialoprotein. IGSF10 depletion in pre-osteoblastic cells attenuated bone sialoprotein expression, suppressed alkaline phosphatase activity, and reduced mineralized nodule formation (Liu et al., 2021). Additionally, several studies have indicated that IGSF10 regulates tumor-cell growth and metastasis and functions as both an immunological indicator and a prognostic marker in several malignancies, including lung adenocarcinoma, breast carcinoma, and gallbladder cancer (Dixit et al., 2022; Zhou et al., 2022; Ling et al., 2024).

In this study, a Chinese patient with delayed puberty, microcephaly, scoliosis, and epilepsy was investigated. Whole-exome sequencing and single-cell RNA sequencing (scRNA-seq) of peripheral blood mononuclear cells (PBMCs) were performed to identify the candidate genetic lesion and generate preliminary hypotheses regarding potential immune-related mechanisms in this patient.

## 2 Materials and methods

### 2.1 Subjects

A Chinese patient presented with delayed puberty, microcephaly, scoliosis and epilepsy was recruited for our hospital. Peripheral blood samples were collected from the patient and her parents. Head computed tomography (CT) and chest X-ray, and laboratory tests, including hormone measurements, were also performed.

### 2.2 Genetic analysis

The DNeasy Blood and Tissue Kit (Qiagen, United States of America) was used to extract the genomic DNA from peripheral blood lymphocytes of family members. The BerryGenomics Biotech Company (Beijing, China) accounts for the whole-exome sequencing and regular filtering analysis of the patient. The data filtering strategy was based on previous studies (Dong et al., 2024; Jiang et al., 2026). The filtered variants were validated and subjected to co-segregation analysis using Sanger sequencing. The primer pairs (the sequence of primers will be provided upon request) were designed by Primer 5. The ABI 3100 Genetic Analyzer was applied to analyze the products of polymerase chain reaction.

### 2.3 Real-time PCR

Peripheral blood samples were collected from the proband and her parents at three separate time points, and total RNA was extracted from peripheral-blood lymphocytes using the PureLink® RNA Mini kit (Thermo Fisher Scientific, 12,183,025). For each time-point RNA sample, 1 μg of RNA was reverse-transcribed into cDNA with the RevertAid First Strand kit (Thermo Fisher Scientific, K1621). Quantitative PCR was run on a Fast 7500 Real-Time PCR System (Applied Biosystems) using Maxima SYBR Green/ROX qPCR Master Mix (2×) (Thermo Fisher Scientific, K0221). Relative gene expression was calculated by the 2^−ΔΔCT^ method. Three independent batches of RNA isolated at different sampling time points from each subject were regarded as biological replicates; each biological replicate contained three technical replicate wells, and the average value of three technical wells was taken as the data of one biological replicate. One-way analysis of variance (ANOVA) followed by *post hoc* pairwise comparison was used for statistical comparisons of IGSF10 expression levels among the proband and parental controls. No multiple testing correction was applied as only one target gene (IGSF10) was detected in this assay.

### 2.4 Single-cell RNA sequencing (scRNA-seq)

Peripheral-blood mononuclear cells from the proband were processed with the Chromium Next GEM Single Cell 3′Kit v3 (10× Genomics) following the vendor’s instructions. Approximately 8,795 cells were combined with 50 µL of barcoded gel beads and 45 µL of partitioning oil on a Chip G to form nanoliter-scale GEMs. Within each GEM, poly(A)+ mRNA underwent reverse transcription, and the resulting full-length, cell-barcoded cDNA was PCR-amplified to prepare sequencing libraries. Library integrity was verified with a Qubit 4.0 Fluorometer and a Qsep100 system (Bioptic, Taiwan); effective concentrations were determined by qPCR. Pools of qualified libraries, normalized by molarity and anticipated yield, were sequenced on an Illumina NovaSeq 6000 using the following configuration: 28 bp Read 1 (16 bp cell/10× barcode +12 bp UMI), 91 bp Read 2 (transcript insert or feature barcode for cell-hashing libraries), and an eight bp i7 sample index. TruSeq Read one and Read two primers enabled standard paired-end sequencing of the single-cell 3′gene-expression libraries.

Using the Chromium Single-Cell Software Suite, we ran the “cellranger count” pipeline to demultiplex libraries and build feature–barcode matrices, aligning reads to the 10× Genomics GRCh38 reference. Multiple samples were pooled with “cellranger aggr” without depth normalization. Resulting UMI count tables were imported into R 4.4.2 and analyzed with Seurat 5.2.1 according to established single-cell best-practice workflows.

### 2.5 The scRNA-seq data analysis

The scRNA-seq dataset of PBMCs from normal individuals whose gender matched that of the proband was downloaded from the GEO database. In this study, the dataset GSE171555 (sequenced by Illumina NovaSeq 6000) was downloaded, and GSM5227117 (female) within it was used as the control scheme for the single-cell data of this study.

The “CreateSeuratObject” command (Seurat v5.1.0) was used to read the data with the parameters set as follows: min. cells = 3 and min. features = 200. In the data cleaning process, the mitochondrial filtering ratio was set at 20%, and the red blood cell filtering ratio was set at 10%. Subsequently, the commands “NormalizeData”, “FindVariableFeatures”, “ScaleData”, and “RunPCA” (Seurat v5.1.0) were executed in sequence. Then, the Harmony method (harmony v1.3.0) was employed to remove batch effects. Finally, the “JoinLayers” command (Seurat v5.1.0) was used to merge the counts and data of multiple samples. According to the results of the ElbowPlot, an appropriate range of dims, which is “1:20″, was selected. The dimensionality reduction methods of tSNE (Seurat v5.1.0) and UMAP were used, and the clustering resolution was set to 0.6. After that, the Immune_All_High.pkl model of Celltypist (v3.3.0) was used to assist in the first annotation and clustering of immune cells. For T cells and B cells, the marker genes and the CellMarker 2.0 database (http://bio-bigdata.hrbmu.edu.cn/CellMarker/) were employed to complete the second-round annotation and clustering of cell subsets. The UMAP method was used for dimensionality reduction again, with the parameter dims set as 1:10 and the resolution set as 0.3. Finally, the correctness of the clustering was confirmed according to the expression levels of cell marker genes. The “FindAllMarkers” function (Seurat v5.1.0) was used to identify differentially expressed genes (DEGs), followed by Gene Set Enrichment Analysis (GSEA) implemented in the clusterProfiler package (v4.12.0) to annotate clusters based on significant DEGs.

Single cell pseudotemporal ordering was performed using the monocle3 package (v1.4.0). The graph_test function was applied to the preprocessed CellDataSet object (cds_test) with the following parameters: neighbor_graph = “principal_graph” (to utilize the principal graph for cell-state transitions) and cores = 8 (to enable parallel computation). This step calculated Moran’s I statistic for each gene, which quantifies the correlation between gene expression patterns and pseudotemporal progression. Significant genes were filtered based on stringent thresholds: genes with a q-value <0.01 (Benjamini-Hochberg-adjusted p-value) were retained. The resulting gene list was then sorted in descending order by Moran’s I values to prioritize genes with strong pseudotemporal dynamics. The top 50 genes exhibiting the highest Moran’s I scores were selected for downstream analysis using the top_n function from the dplyr package (v1.1.4).

### 2.6 Visualization and statistical analysis

GraphPad Prism (version 10.1.2) and R (version 4.4.2) may be used for the visualization of experimental results. GraphPad Prism, which was used for drawing bar charts, box plots and violin plots with a small amount of data. It can perform built-in T-tests, Anova tests, etc., and mark the asterisks (*) or P values. R, which was used for dealing with large amounts of data or complex graphing. R packages such as ggplot2 and dplyr are used for the drawing and combination of graphs.

## 3 Results

### 3.1 Clinical description of a patient with delayed puberty, microcephaly and epilepsy

**FIGURE 1:**
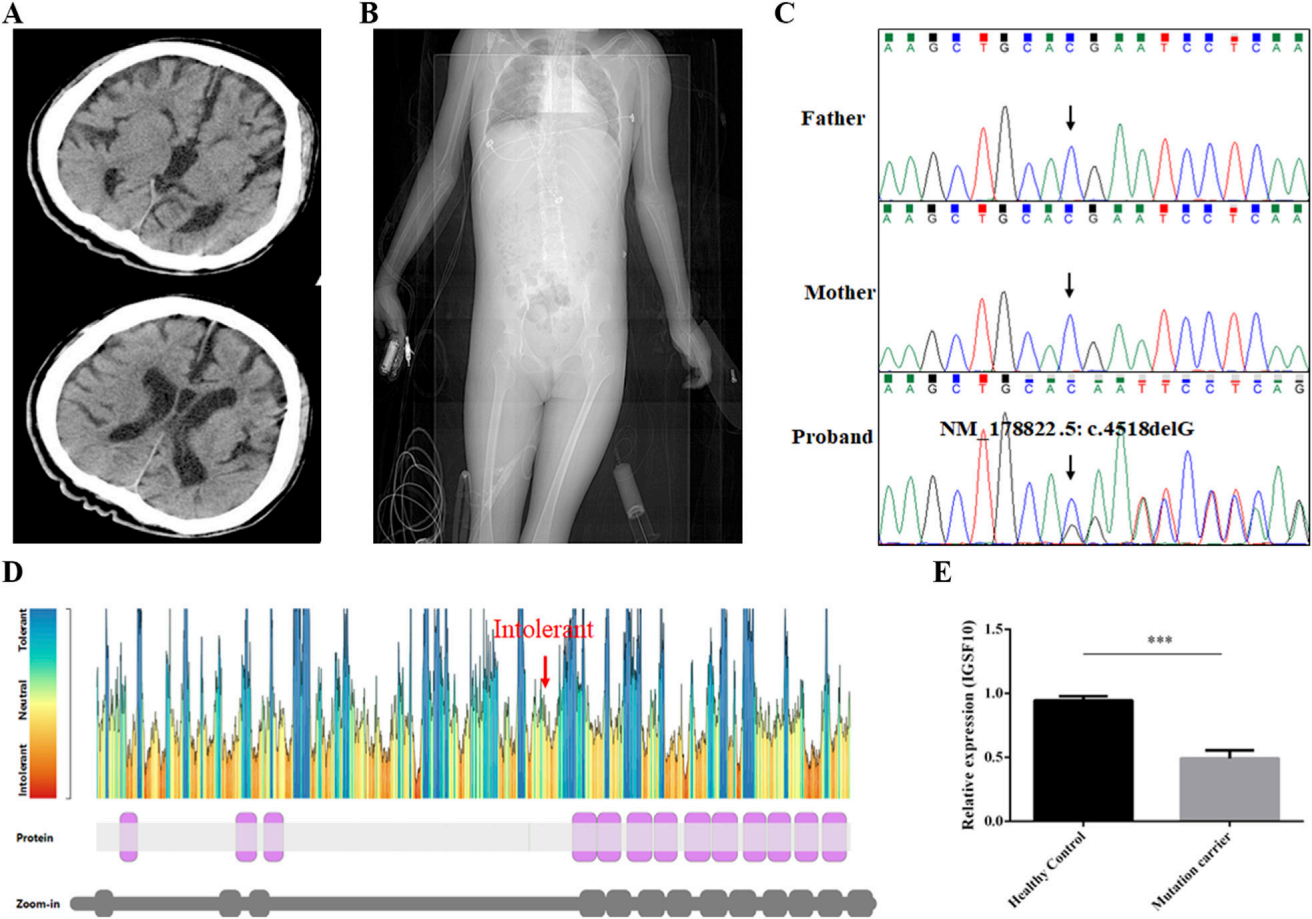
The clinical information and genetic analysis of the proband. **(A)** The head CT of the proband. **(B)** The chest X-ray of the proband. **(C)** Sanger sequencing chromatogram demonstrates the heterozygosity for an *IGSF10* deletion mutation (NM_178,822.5: c.4518delC; p. H1506Qfs*13) in the proband. **(D)** The tolerance analysis of mutant amino acid sites of IGSF10 was predicted by MetaDome software. **(E)** Mean relative IGSF10 expression (±SEM) based on three independent biological replicates from serial blood sampling at different time points; one-way ANOVA followed by *post hoc* test was used for intergroup comparison. ***P < 0.001.

The proband, a 20-year-old female, was admitted to the hospital because of primary amenorrhea. Medical history revealed that she was born at term and had brain developmental delays. Learning and speech difficulties were reported. Head CT showed microcephaly, with slightly enlarged ventricles, sulci, and cisterna (Figure 1A). Chest X-ray showed S-shaped lateral curvature of the spine (Figure 1B). Pubertal development was reported to have started at approximately 16–17 years of age. She also had a history of epilepsy and had been hospitalized for recurrent status epilepticus and pneumonia during the previous 1 year. Laboratory tests showed low serum levels of Prolactin (2.5 ng/mL), Estradiol (9 pg/mL), Luteinizing hormone (0.22 mIU/mL) and Follicle stimulating hormone (1.4 mIU/mL). Progesterone and testosterone levels were normal (Supplementary Table S1). Detailed clinical information of the proband and her parents is shown in Supplementary Table S2.

### 3.2 Genetic analysis identified a *de novo* mutation of IGSF10 in the patient

Karyotype analysis showed that the proband was normal (46, XX). Whole-exome sequencing was performed to identify the underlying genetic defect. A total of 79,576 variants were detected in the proband. After data filtering, 10 candidate variants remained, as detailed in Supplementary Table S3. Validation by Sanger sequencing and co-segregation analysis revealed a *de novo* IGSF10 variant (NM_178,822.5: c.4518delC; p. H1506Qfs*13), as a potential genetic lesion in the patient (Figure 1C). This variant generates a premature termination codon at position 1519 in exon 6 of IGSF10 and is predicted to result in protein truncation. MetaDome predicted that the His1506 residue is located in the intolerant region of IGSF10 (Figure 1D). The variant was also predicted to trigger IGSF10 mRNA degradation in the mutation carrier, consistent with nonsense-mediated decay (Carrard and Lejeune, 2023). Accordingly, RNA was extracted from the mononuclear cells from the proband and healthy controls, namely, her parents. With control expression normalized to 1, real-time PCR revealed an approximately ∼57% reduction in IGSF10 transcript levels in the proband (Figure 1E).

### 3.3 scRNA-seq analysis revealed altered peripheral blood immune-cell composition

**FIGURE 2:**
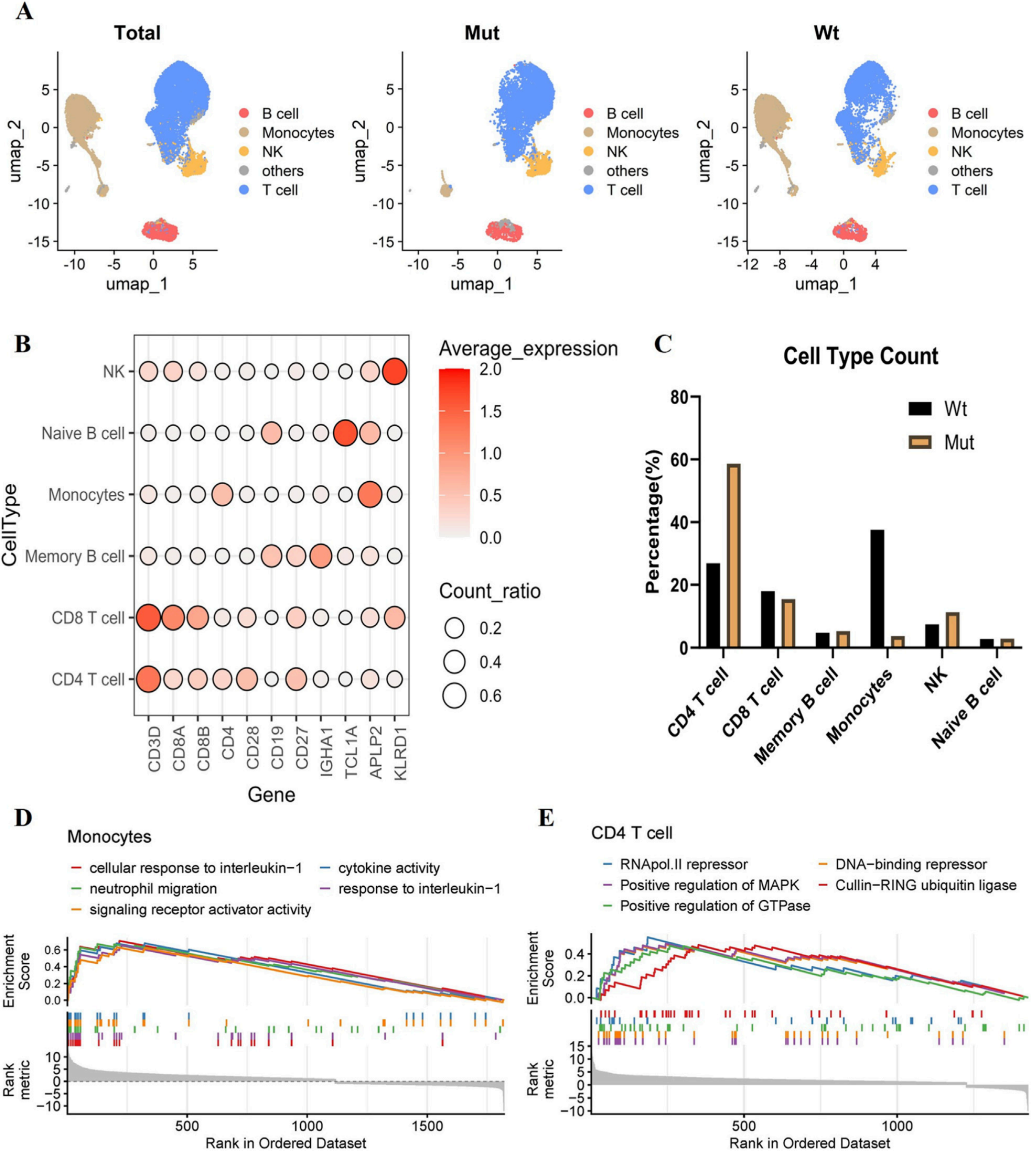
Single-cell RNA-seq profiling of PBMCs reveals altered immune cell composition in the proband. **(A)** Uniform Manifold Approximation and Projection (UMAP) visualization of PBMCs from the IGSF10 mutation carrier (Mut) and the healthy control (Wt). Six major cell types were identified (CD4^+^ T cell, CD8^+^ T cell, Memory B cell, Naive B cell, Monocytes, NK cell). Total: Overall, Mut: Proband (mutant type), Wt: Normal individuals. **(B)** The expression of marker genes in each cell population demonstrated the correctness of cell clustering. The shade of color represented the average expression level, and the size of the circle represented the proportion of the number of cells expressing the gene. **(C)** Bar plot showing the relative proportion of each cell type in total captured cells for the Mut and Wt groups. Detailed cell counts and percentages are provided in Supplementary Table S4. There were obvious changes in CD4^+^ T cells and Monocytes. **(D,E)** Gene Set Enrichment Analysis (GSEA) annotations that were significantly upregulated in the proband compared to the normal group for the 2 cell lines, CD4^+^ T cells and Monocytes, whose numbers had changed. The line graph in the upper part represented the Running Enrichment Score; the middle graph illustrated the genes; and the lower graph showed the ranking of gene expression levels.

To explore the immune correlates of the novel IGSF10 mutation, we performed scRNA-seq on PBMCs from the proband. Peripheral blood cell populations were identified and clustered by integrating Celltypist, CellMarker 2.0, and cell-marker genes (Figures 2A,B; Supplementary Figures S1, S2). Six major cell populations were identified, including CD4^+^ T cells, CD8^+^ T cells, naïve B cells, memory B cells, monocytes, and natural killer (NK) cells; the remaining rare cell populations were classified into the “others” group (Figures 2A,B). IGSF10 expression was examined across all PBMC subsets. Low and scattered expression was observed in CD4^+^ T cells, monocytes, and other immune populations (Supplementary Figure S4). The proportion of CD4^+^ T cells among total blood cells in the proband carrying the IGSF10 variant was much higher than that in healthy individuals. In contrast, the proportion of monocytes was markedly reduced. The proportion of CD8^+^ T cells decreased slightly, the proportion of NK cells increased slightly, and no substantial changes were observed in the proportions of other cell types (Figure 2C).

To further explore the functional implications of the altered cell-type proportions, Gene Set Enrichment Analysis (GSEA) was performed. Ubiquitin ligases, DNA-binding transcription factors, GTPases, and the MAPK signaling pathway were significantly upregulated in CD4^+^ T cells from the proband (Figure 2D). In monocytes, pathways related to IL-1 response, cytokine signaling, signal receptor activity, and neutrophil migration were prominently activated (Figure 2E).

The top 20 significantly upregulated genes in the CD4^+^ T cells and monocytes of the proband were analyzed. Four genes were significantly upregulated in both cell types: *SLC35F1*, LINC00486, AC068282.1, and AC022217.3. Among these, only SLC35F1 was a coding RNA and showed the most significant upregulation (Supplementary Figure S1). *BTBD11* was expressed only in CD4^+^ T cells, whereas *ALDH1A2, IL1A, CCL20,* and *TNFAIP6*, were specifically expressed only in monocytes (Supplementary Figure S1).

### 3.4 scRNA-seq analysis identifies alterations in CD4^+^ T cell subsets

**FIGURE 3:**
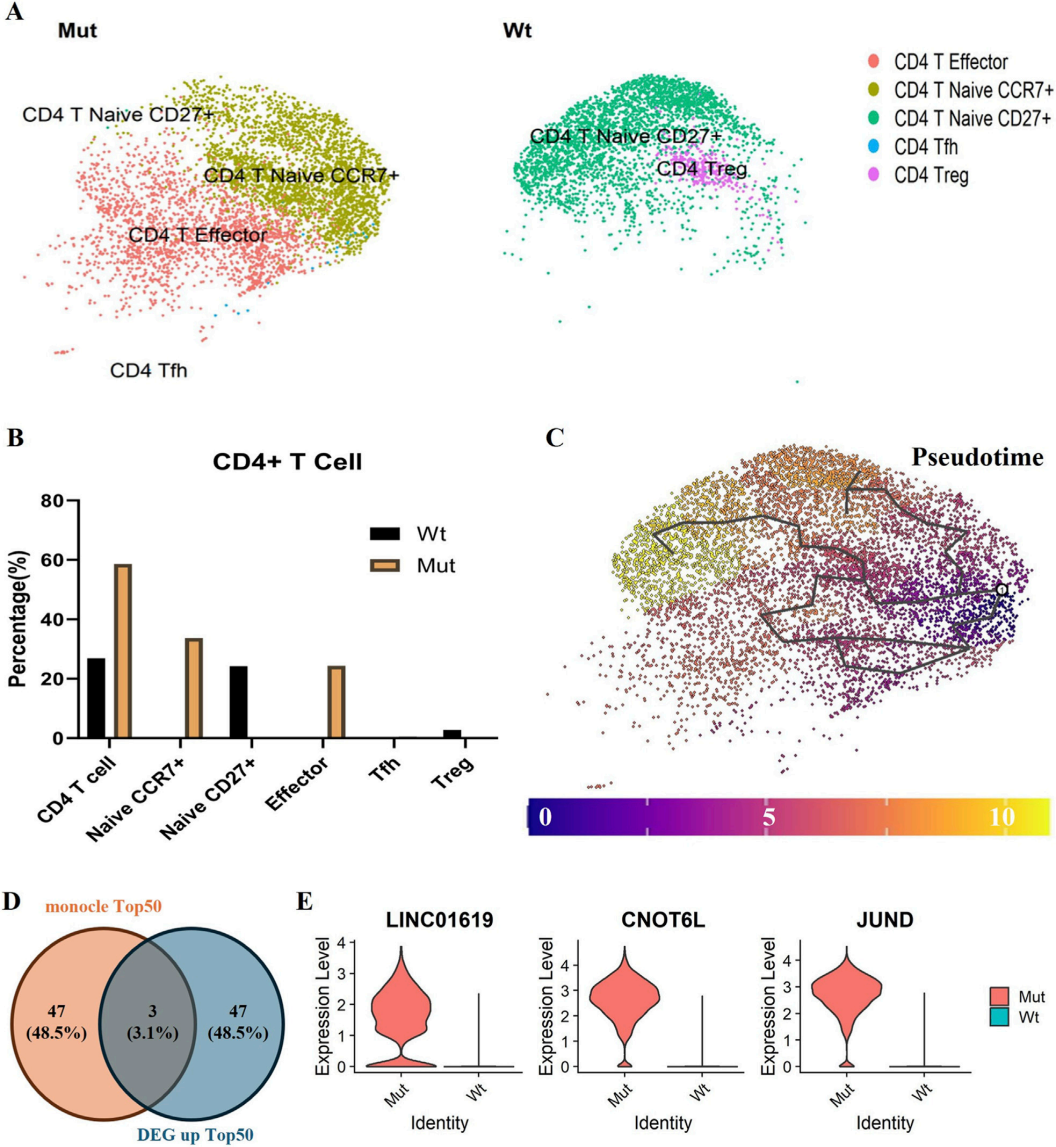
Identification of hub genes associated with CD4^+^ T cell alterations in the proband. **(A)** UMAP was used to display the annotation results of cell subsets, distinguishing five subsets (CD4^+^ T Effector, CD4^+^ T Naive CCR7+, CD4^+^ T Naive CD27^+^, CD4^+^ Tfh, CD4^+^ Treg). Mut: Proband (mutant type), Wt: Normal individuals. **(B)** The bar chart showed the percentage of each cell subtype relative to the total number of cells in the proband or the normal group. **(C)** Pseudotime analysis was conducted based on UMAP, with black lines marking the possible cell differentiation pathways. **(D)** Venn diagram showing the intersection of the top 50 genes with the strongest pseudotime correlation and the top 50 significantly upregulated genes in the proband; three hub genes were identified. **(E)** The violin plot showed the comparison of the expression levels of the hub genes in the CD4^+^ T cells of the proband and those of normal individuals.

Subcluster characterization of CD4^+^ T cells identified effector T cells as the predominant contributor to the observed CD4^+^ T cell elevation. Notably, naive T cells in healthy controls displayed a CD27^+^ “activation-primed” state, whereas those from the proband predominantly exhibited a CCR7+ “quiescent” signature (Figures 3A,B). Pseudotime trajectory reconstruction uncovered two divergent differentiation axes: (1) a prominent pathway driving quiescent naive T cells toward terminal effector T cells differentiation, and (2) a minor pathway redirecting quiescent naive T cells toward activated naive states (Figure 3C).

To systematically identify molecular drivers, we employed an integrative analytical framework: (1) Top 50 genes ranked by pseudotemporal correlation strength (Moran’s I) and (2) Top 50 differentially expressed genes (log 2 Fold Change) were intersected to pinpoint trajectory-critical regulators (Figure 3D). The data showed that CD4^+^ T cell: *CNOT6L* (chromatin modifier), *JUND* (AP-1 transcription factor), and LINC01619 (non-coding RNA) were identified as candidate regulators potentially associated with effector T cell differentiation (Figure 3E).

### 3.5 The scRNA-seq analysis identifies monocyte subset alterations

**FIGURE 4:**
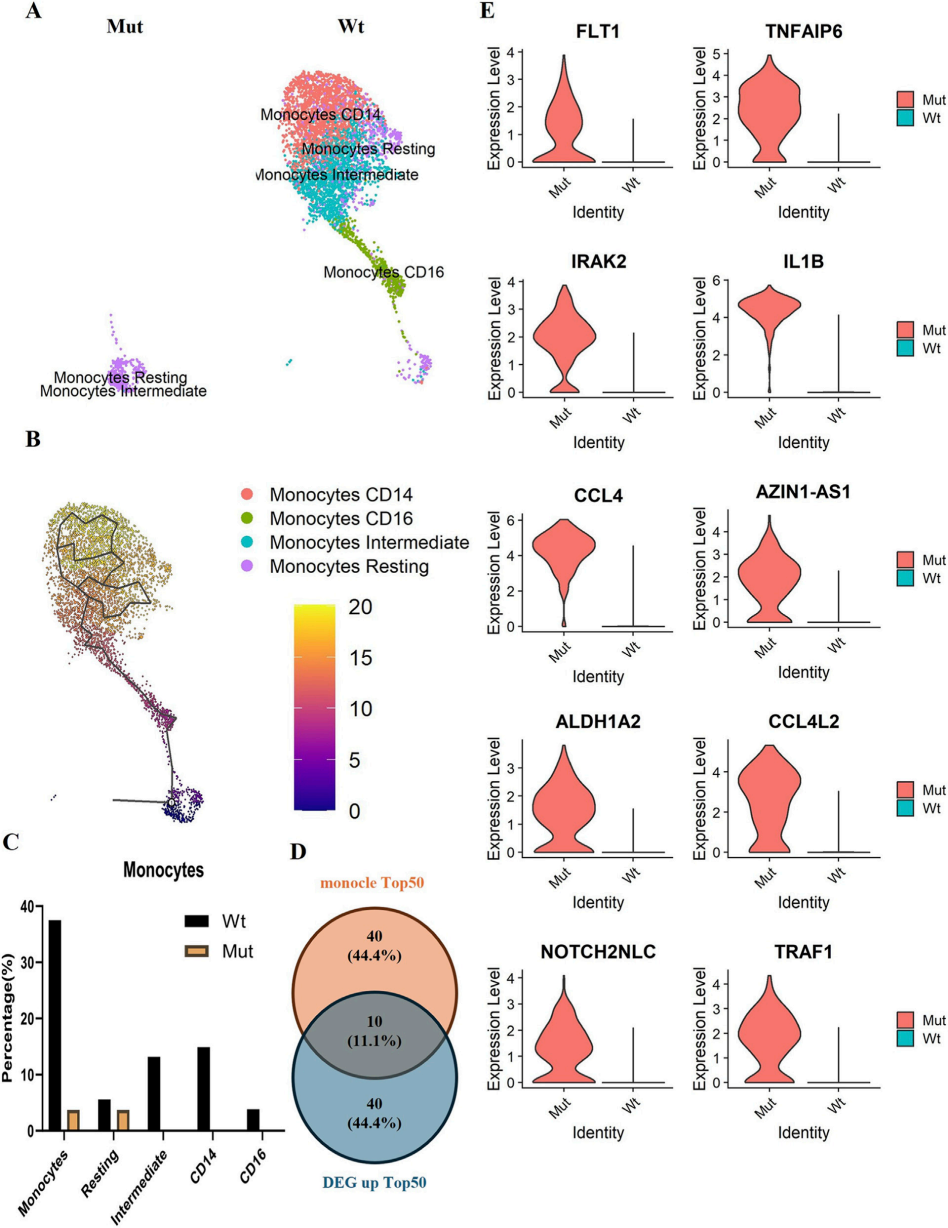
Identification of core genes associated with monocyte alterations in the proband. **(A)** UMAP was used to display the annotation results of cell subsets, distinguishing four subsets (Monocytes CD14^+^, Monocytes CD16^+^, Monocytes Intermediate, Monocytes Resting). Mut: Proband (mutant type), Wt: Normal individuals. **(B)** The bar chart showed the percentage of each cell subtype relative to the total number of cells in the proband or the normal group. **(C)** Pseudotime analysis was conducted based on UMAP, with black lines marking the possible cell differentiation pathways. **(D)** Venn diagram showing the intersection of the top 50 genes with the strongest pseudotime correlation and the top 50 significantly upregulated genes in the proband; ten core genes were identified. **(E)** The violin plot showed the comparison of the expression levels of the core genes in the Monocytes of the proband and those of normal individuals.

Monocyte profiling revealed a marked reduction in total monocyte counts in the proband, with resting monocytes constituting >99% of the population (Figure 4A). Pseudotime trajectory analysis delineated potential phenotypic conversion routes among monocyte subsets, suggesting that resting monocytes in healthy individuals’ differentiation into CD14^+^ classical, CD16^+^ non-classical, and intermediate subtypes (Figure 4B). However, this differentiation appeared substantially impaired in the proband (Figure 4C). An analytical strategy similar to that used for CD4^+^ T cells was applied to identify potential molecular drivers (Figure 4D). Key genes, including *CCL4*/*CCL4L2* (chemotactic signaling), *IRAK2*/*TRAF1* (NF-κB activation), and *IL1B*/*TNFAIP6* (pro-inflammatory mediators), ALDH1A2 (retinoic acid synthesis) and NOTCH2NLC (NOTCH pathway modulator) were identified as candidate molecules potentially involved in monocyte phenotypic conversion (Figure 4E).

## 4 Discussion

To investigate the genetic basis of delayed puberty, microcephaly, scoliosis, and epilepsy in the patient, whole-exome sequencing was performed, and a *de novo* frameshift variant in IGSF10, NM_178,822.5:c.4518del (p.His1506Gln fs Ter13), was identified. This alteration was absent from 200 ethnically matched local controls and from dbSNP155, 1000 Genomes and gnomAD. Real-time PCR further provided that the mRNA level of IGSF10 was much lower in mutation carrier than in health controls. PBMC scRNA-seq suggested that the number of effector T cells was markedly increased, whereas the number of monocytes was markedly decreased, in the mutation carrier. Further analysis identified three key regulatory genes including *CNOT6L*, *JUND* and LINC01619 in governing effector T cells differentiation, and 10 hub genes involving *CCL4*, *CCL4L2*, *IRAK2*, *TRAF1*, *IL1B*, *TNFAIP6*, *ALDH1A2* and NOTCH2NLC in regulating monocyte phenotypic conversion. This study represents the first comprehensive single-cell mapping of PBMCs in an IGSF10 mutation carrier, providing preliminary observational data and testable hypotheses for delayed puberty associated with IGSF10 mutations.

The IGSF10 protein contains an N-terminal leucine-rich repeat domain and 12 Ig-like domains and is involved in guiding GnRH neurons from the nasal placode to the hypothalamus during embryonic development (Budny et al., 2020; Rao et al., 2026). Three previous studies have identified 10 *IGSF10* mutations in patients with delayed puberty, pituitary hormone deficiencies and amenorrhea (Budny et al., 2020; Damon et al., 2024). One study suggested that *IGSF10* mutation may lead to cleidocranial dysplasia (Liu et al., 2021), a skeletal disorder (Supplementary Figure S3). In the present case, a novel IGSF10 variant, NM_178,822.5:c.4518del (p.His1506GlnfsTer13), was identified in a Chinese patient with delayed puberty, amenorrhea, microcephaly, and epilepsy. According to ACMG guideline (Richards et al., 2015), this variant was classified as pathogenic based on the following criteria: PVS1 + PS2 + PM2 + PM4 + PP + PP3. This case expands the reported mutational spectrum of IGSF10 deficiency and suggests that the phenotypic spectrum may extend to microcephaly, scoliosis, and epilepsy; these novel phenotypic associations require replication in independent patient cohorts.

scRNA-seq revealed that the novel mutation was associated with dramatic changes in effector T cell and monocyte numbers in the proband. Effector T cells can directly attack the ovarian tissue or affect the ovarian blood supply, leading to ovarian failure and amenorrhea (Liu et al., 2025). Abnormal activation of effector T cells and reduction of monocytes may affect normal function of the hypothalamic-pituitary-ovarian axis and may induce inflammation of the hypothalamus or pituitary gland, leading to abnormal production of gonadotropin-releasing hormone or gonadotropins, which in turn may affect ovarian function and contribute to delayed puberty (Tulic et al., 2012; Hong et al., 2020; Younesi et al., 2021). Monocytes mainly contribute to immune surveillance. Reduced monocyte numbers may impair immune surveillance and increase susceptibility of the brain to infection or inflammation, which may trigger seizures (Cugurra et al., 2021). The increased mRNA levels of several hub genes, including *IRAK2*, *TRAF1*, *IL1B* and *TNFAIP6*, in the proband suggested an inflammatory response. These findings provide preliminary evidence that IGSF10 deficiency may be associated with systemic inflammatory alterations, which could hypothetically contribute to amenorrhea, cerebral dysplasia, and seizures; this causal relationship remains to be verified.

In the proband, *CNOT6L*, *JUND* and LINC01619 were prioritized as nodal regulators potentially involved in effector T-cell differentiation. This bifurcation suggests sustained effector commitment in the proband’s immune milieu, accompanied by homeostatic replenishment of quiescent naïve T cells, which may represent an adaptation to persistent immune stimulation. Mechanistically, the inverted CCR7/CD27 axis in the proband’s naive T cells points to defective T cell receptor (TCR) signaling dynamics or disrupted cytokine gradients (Matsuki et al., 2014), essential regulators of T cell homeostasis. Pathologically expanded effector T cells populations likely mediate tissue-specific inflammation and autoimmune pathology, which further disrupt the function of brain and hypothalamic-pituitary-ovarian axis (Dikiy and Rudensky, 2023). Our study revealed that as a member of immunoglobulin superfamily, IGSF10 may be involved in regulating T cell differentiation, which could potentially influence brain development via immune-mediated pathways.

Single-cell RNA-seq also suggested that the differentiation of CD14^+^ classical, CD16^+^ non-classical, and intermediate subtypes was impaired in the proband. Such developmental blockades may act together with the dysregulated T cell compartment and contributing to impaired immune surveillance and chronic inflammation through two synergistic mechanisms (Lee et al., 2020): (1) failure to generate pro-inflammatory CD16^+^ monocytes may limit antigen-presentation capacity, and (2) accumulated resting monocytes may exacerbate T cell exhaustion. These findings indicate that IGSF10 may participate in monocyte differentiation and that monocyte dysfunction may indirectly contribute to the patient’s reproductive, neurological, and skeletal phenotypes. Although IGSF10 showed low transcript abundance in mature circulating immune cells, this expression pattern does not exclude a critical cell-intrinsic regulatory function during early immune progenitor differentiation in primary lymphoid organs, the primary developmental window in which IGSF10 may exert its biological effects. However, whether the immune alterations are primary, as a direct effect of IGSF10 deficiency disrupting immune progenitor differentiation, or secondary to chronic hormonal and neurological dysfunction cannot be distinguished in this study and requires further validation.

In addition, skull and spine developmental abnormalities were observed in the proband. Previous work demonstrated that IGSF10 silencing markedly reduces bone sialoprotein (BSP) expression (Liu et al., 2021). BSP belongs to the SIBLING family of extracellular-matrix glycoproteins enriched in mineralized tissues (Maalouf et al., 2022) and has long served as an early indicator of osteoblast commitment, rising in tandem with the onset of matrix mineralization (Chavez et al., 2023). It is therefore proposed that the frameshift deletion in IGSF10 reduced gene expression at the mRNA level, which may have downregulated BSP and contributed to the skull and spinal defects observed in the patient. This finding further supports a role for IGSF10 in skeletal development.

Several limitations of this study should be acknowledged. First, because of technical constraints associated with the 8562 bp full-length IGSF10 transcript, including difficulties in full-length coding-sequence cloning and the lack of validated specific antibodies, the effect of the frameshift variant was not validated at the protein level, and *in vitro* functional assays were not performed in cell models. These experiments will be prioritized in follow-up studies. Second, the immune findings were derived from single-cell transcriptomic profiling of peripheral blood from a single patient. The direct causal relationship between the observed effector T-cell and monocyte perturbations and the patient’s multisystem clinical phenotypes remains to be functionally verified. These findings provide preliminary evidence and mechanistic hypotheses regarding the immunological role of IGSF10, which require further validation in larger patient cohorts and targeted experimental models.

In summary, a novel IGSF10 deletion, NM_178,822.5:c.4518del (p.His1506Gln fs Ter13), was identified by whole-exome and Sanger sequencing in a Chinese patient with delayed puberty, microcephaly, scoliosis, and epilepsy. PBMC scRNA-seq analysis revealed that the variant was associated with marked alterations in effector T-cell and monocyte numbers. Further analysis suggested that the novel variant may affect differentiation of T cells and monocytes, which may contribute to amenorrhea, cerebral dysplasia, and seizures. In addition, scoliosis was identified as a rare phenotype in an IGSF10 mutation carrier. This case expands the reported mutational spectrum of IGSF10 deficiency and suggests scoliosis and epilepsy as rare potential phenotypic manifestations. These findings generate preliminary hypotheses regarding the interplay among IGSF10, immune function, hormone secretion, and brain and skeletal development, which require validation in larger patient cohorts.

## Data Availability

The datasets presented in this study can be found in online repositories. The names of therepository/repositories and accession number(s) can be found below: https://ngdc.cncb.ac.cn/search/specific?db=hra&q=HRA016149, China National Center for Bioinformation (HRA016149).
