# Supplementary material for: Clinical and immunological features of a patient exhibiting delayed puberty, microcephaly, scoliosis and epilepsy caused by a novel mutation in IGSF10
Source: Front Cell Dev Biol. 2026 Jul 30;14:1816964. doi: 10.3389/fcell.2026.1816964 (PMC13469598; doi:10.3389/fcell.2026.1816964)
Supplement: Supplementary file 1 [file DataSheet1.docx]

Figure S1. The marker genes of the CD4 T cells.

Figure S2. The marker genes of the monocytes.

Figure S3. The summary of reported IGSF10 mutations. The black squares indicated patients with delayed puberty. The orange square suggested patients with cleidocranial dysplasia. The red square indicated the present study.

Figure S4. High-resolution UMAP clustering and IGSF10 expression profile in PBMCs. **A.** High-resolution UMAP plots show all cell subset clustering in peripheral blood mononuclear cells from the healthy control (Wt) and the IGSF10 mutation carrier (Mut). **B.** UMAP plots display cells with detectable IGSF10 transcript (blue) overlaid on all captured cells (gray) in the two groups. IGSF10 shows low and scattered expression across peripheral immune subsets in both groups. Detailed cell counts and proportions of each subset are provided in Table S4.

Table S1. The laboratory testing of gonadal hormone in the proband.

| **subjects** | **Test value** | **Reference range** |
| --- | --- | --- |
| Prolactin | 2.50 | 3–29 ng/mL |
| Estradiol | 9 | Early follicular: 10–50 pg/mL  Late follicular: 60–200 pg/mL  Midcycle 120–375 pg/mL  Luteal phase: 50–260 pg/mL  Post menopausal: <20 pg/mL |
| Progesterone | 0.21 | Follicular: 0.2–1.6 ng/mL  Luteal phase: 3–22 ng/mL  Mid-luteal phase: 5–22 ng/mL  Over 60 years old: 0.2–0.6 ng/mL |
| Luteinizing hormone | 0.22 | Follicular: 2–15 mIU/mL  Midcycle: 22–105 mIU/mL  Luteal phase: 0.1–19 mIU/mL  Post menopausal: 16–64 mIU/mL |
| Testosterone | 0.12 | 0.1–0.7 ng/mL |
| Follicle stimulating hormone | 1.4 | Follicular: 2–12 mIU/mL  Midcycle: 5–24 mIU/mL  Luteal phase: 1.5–10 mIU/mL  Post menopausal: 21–131 mIU/mL |

Table S2. The detailed clinical data of each individual in this study.

| No. | Gender | Age | CT | X-ray | Laboratory testing |
| --- | --- | --- | --- | --- | --- |
| 1  (Proband’s father) | Male | 43 | Normal | Normal | Prolactin (4.3 ng/mL) normal  Estradiol (31 pg/mL) normal  Progesterone (0.4 ng/mL) normal  Luteinizing hormone (6.9 mIU/mL) normal  Testosterone (0.38 ng/mL) normal  Follicle stimulating hormone (4.2 mIU/mL) normal |
| 2  (Proband’s mother) | Female | 42 | Normal | Normal | Prolactin (20 ng/mL) normal  Estradiol (60 pg/mL) normal  Progesterone (3.4 ng/mL) normal  Luteinizing hormone (7.41 mIU/mL) normal  Testosterone (0.49 ng/mL) normal  Follicle stimulating hormone (9.61 mIU/mL) normal |
| 3  (Proband) | Female | 20 | microcephaly, with slightly enlarged ventricles, sulci and cisterna | S-lateral curvature of the spine | Prolactin (2.50) <3 ng/mL  Estradiol (9) <10 pg/mL  Progesterone (0.21 ng/mL) normal  Luteinizing hormone (0.22) <2 mIU/mL  Testosterone (0.12 ng/mL) normal  Follicle stimulating hormone (1.4) <2 mIU/mL |

Table S3. The mutation list of Sanger sequencing validation and co-segregation analysis.

| No. | Gene | Position | RB | AB | Transcript | Variant | OMIM |
| --- | --- | --- | --- | --- | --- | --- | --- |
| 1 | MUTYH | chr1:45334493-45334493 | G | A | NM_001128425.2 | c.55C>T | AR. Adenomas |
| 2 | ACBD5 | chr10:27219804-27219804 | C | T | NM_145698.5 | c.544G>A | AR. Retinal dystrophy with leukodystrophy |
| 3 | ESRRA | chr11:64315821-64315821 | G | T | NM_004451.5 | c.1127G>T | Anorexia nervosa/bulimia nervosa |
| 4 | ESRRA | chr11:64315848-64315848 | T | C | NM_004451.5 | c.1154T>C | Anorexia nervosa/bulimia nervosa |
| 5 | COL4A2 | chr13:110489787-110489787 | T | C | NM_001846.4 | c.3346+2T>C | AD. Brain small vessel disease |
| 6 | MEFV | chr16:3249468-3249468 | C | T | NM_000243.3 | c.1223G>A | AD. Familial Mediterranean fever |
| 7 | MEFV | chr16:3249586-3249586 | G | A | NM_000243.3 | c.1105C>T | AD. Familial Mediterranean fever |
| 8 | SERPINB7 | chr18:63793279-63793279 | T | G | NM_003784.4 | c.336+2T>G | AR. Palmoplantar keratoderma |
| 9 | IGSF10 | chr3:151445463-151445463 | G | - | NM_178822.5 | c.4518delC | AD. Delayed puberty |
| 10 | SLC26A4 | chr7:107683453-107683453 | A | G | NM_000441.2 | c.919-2A>G | AR. Deafness |

Chr, Chromosome; POS, position; RB, reference sequence base; AB, alternative base identified; AD, autosomal dominant; AR, autosomal recessive.

Table S4

| group | SubCellType | count | percentage |
| --- | --- | --- | --- |
| Mut | CD4 T cell | 3699 | 44.61464 |
| Mut | CD8 T cell | 2354 | 28.39223 |
| Mut | ILC | 932 | 11.2411 |
| Mut | Megakaryocyte | 307 | 3.70281 |
| Mut | Memory B cell | 411 | 4.957182 |
| Mut | Monocytes | 306 | 3.690749 |
| Mut | Naive B cell | 230 | 2.774092 |
| Mut | others | 52 | 0.627186 |
| Wt | CD4 T cell | 2801 | 38.11403 |
| Wt | CD8 T cell | 699 | 9.511498 |
| Wt | ILC | 514 | 6.994149 |
| Wt | Megakaryocyte | 12 | 0.163288 |
| Wt | Memory B cell | 362 | 4.92584 |
| Wt | Monocytes | 2083 | 28.34399 |
| Wt | Naive B cell | 764 | 10.39597 |
| Wt | others | 114 | 1.551231 |
